# Getting PIKy with the lysosome membrane (again)

**DOI:** 10.1080/27694127.2024.2412916

**Published:** 2024-10-12

**Authors:** Alison D. Klein, Michael Overholtzer

**Affiliations:** a BCMB Graduate Program, Weill Cornell Medical College, New York, NY, USA; b Cell Biology Program, Memorial Sloan Kettering Cancer Center, New York, NY, USA; c Gerstner Sloan Kettering Graduate School of Biomedical Sciences, Memorial Sloan Kettering Cancer Center, New York, NY, USA

**Keywords:** ATG8, autophagy, CASM, LC3, lysosome, microautophagy, PIKfyve, TFEB, TRPML1

## Abstract

Much is still unknown about microautophagy and its regulators. In our recent paper, one such regulator of microautophagy, the lipid kinase PIKfyve, is described. Previously it was found that treating cells with agents like lysomotropic drugs or proton ionophores, which alter lysosomal osmotic potential and pH, leads to a form of microautophagy that selectively degrades transmembrane proteins. Induction of this type of microautophagy is linked to a lysosomal stress response that involves the targeting of macroautophagy proteins, like ATG8s, to the lysosome membrane, through a mechanism called CASM. We found that CASM-induced microautophagy turns over ATG8s and other lysosomal membrane proteins, and requires PIKfyve activity functioning downstream of ATG8 lipidation. The lysosome biogenesis transcription factor TFEB is induced in parallel to microautophagy, in a CASM-dependent, but PIKfyve-independent manner. These findings demonstrate that stressors that engage CASM cause selective turnover by microautophagy that is coordinated with lysosome biogenesis through a mechanism that is separable through PIKfyve.

## Punctum

Lysosomal stress responses are key regulators of cellular fitness that control the biogenesis of new lysosomes, or control the repair, turnover, and exocytosis of lysosomes that are stressed or damaged. A failure of cells to maintain healthy lysosome networks is thought to contribute to aging and the development of Parkinson’s and other neurodegenerative diseases or neurodevelopmental disorders. Understanding how cells can repair or eliminate damaged lysosomes to maintain lysosomal homeostasis is therefore critical to gain new insights into a variety of human pathologies.

Our lab developed an assay to measure lysosome membrane turnover by tagging lysosomal transmembrane proteins with GFP and monitoring the appearance of free GFP by western blotting, an approach used previously to quantify lysosomal turnover of autophagy substrates in yeast and mammalian cells. GFP resists degradation by cathepsins and thus turns over more slowly than the transmembrane proteins to which it is tagged, resulting in the appearance of a free GFP band that is indicative of lysosomal digestion. This approach led us to discover that lysosomal membrane turnover occurs in response to treatment with stressors that induce the lipidation of ATG8s (encoded by *Autophagy-related gene 8s or ATG8s*) onto lysosomal membranes, an activity referred to as Conjugation of ATG8s onto Single Membranes (CASM). CASM occurring at the lysosomal membrane is induced in response to abnormal elevations in lysosomal pH or osmotic stress, and is distinguishable from macroautophagy by numerous criteria, sometimes referred to as the “CASM toolkit”, including its independence of regulation by the ULK kinase complex or ATG9A; its dependence on activity of the vacuolar-type ATPase (v-ATPase) and particular residues in ATG16L1 that are dispensable for macroautophagy; and its unique morphological appearance involving direct conjugation of ATG8s onto single membranes. This form of regulation of CASM is also referred to as v-ATPase-ATG16L1-induced LC3 lipidation (VAIL). Curiously, we uncovered that lysosomal membrane turnover induced by CASM occurs in a selective manner, as some but not all transmembrane proteins were observed to undergo degradation. We also found that selective turnover involves the formation of intraluminal vesicles (ILVs) derived from the limiting lysosomal membrane, an activity called microautophagy.

To further study this type of microautophagy in our recent paper,^1^ it became necessary to develop a microscopy-based approach that would allow us to image with enhanced spatiotemporal resolution the composition of ILVs, which are small in size and move rapidly within the fluid-filled environment of the lysosome lumen. To image microautophagy, we co-expressed fluorescently-tagged versions of TRPML1 (Transient Receptor Potential Cation Channel, Mucolipin Subfamily, Member 1) and LAMP1 (Lysosome-Associated Membrane Protein 1), choosing these transmembrane proteins because one (TRPML1) was found to undergo turnover under CASM-inducing conditions while the other (LAMP1) was not. We reasoned that the simplest model to explain selective degradation might be the exclusion of proteins that resist degradation from the formation of ILVs. Surprisingly, we found that the ILV populations induced by CASM contained both LAMP1 and TRPML1 at the same frequency, and although composition of the ILVs themselves could differ, populations of ILVs positive for both proteins were found within the same lysosome. This observation suggests that selectivity for turnover is unlikely to be dictated by the process of ILV formation itself, but rather by some downstream component like ILV composition.

We also discovered that ATG8s turn over during CASM-induced microautophagy, a conclusion supported by both imaging and western blotting experiments using a GFP-tagged LC3 (Microtubule-Associated Protein 1 Light Chain 3). We also identified a key regulator of this type of microautophagy, the lipid kinase PIKfyve (*Phosphoinositide Kinase, FYVE-Type Zinc Finger Containing*), which catalyzes the formation of phosphatidylinositol (3,5)-bisphosphate (PI(3,5)P_2_) on endolysosomal membranes, and here, found to be required for efficient ILV formation and lysosomal membrane protein turnover. Our study also implicated TRPML1 acting downstream of PIKfyve to support the formation of ILVs, suggesting that cation fluxes may be linked to this process. While PIKfyve was required for the appearance of LC3-positive ILVs, its inhibition did not reduce the lipidation of LC3 onto lysosomal membranes, revealing that PIKfyve regulates microautophagy by acting downstream of CASM to support ILV formation.

We further examined this pathway in the context of lysosome biogenesis, as the activity of a key lysosome biogenesis transcription factor, TFEB (Transcription Factor EB), was recently shown to be activated in response to CASM. We indeed found that TFEB was translocated into the nucleus in parallel to the induction of microautophagy, in a CASM-dependent, but PIKfyve-independent manner. These findings indicate that lysosome biogenesis and microautophagy are induced in parallel, downstream of CASM but are regulated by separable mechanisms.

**Figure 1.**
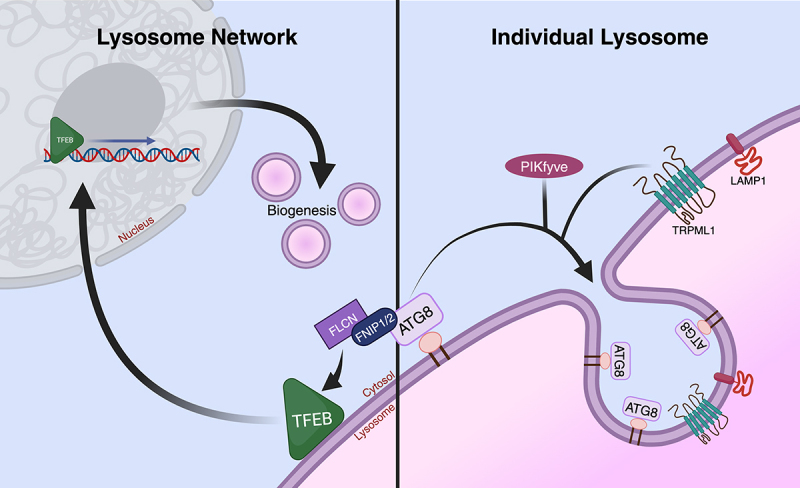
Lysosomal stress-induced microautophagy and lysosome biogenesis can be regulated by CASM. Left: CASM occurring at the lysosomal membrane activates the lysosome biogenesis transcription factor TFEB in an FNIP1/FNIP2-Folliculin dependent manner. Right: CASM induces a type of microautophagy involving invagination of the limiting lysosomal membrane, an activity that forms ILVs and leads to selective turnover of transmembrane proteins. CASM has the dual ability of targeting individual lysosomes for turnover (right) and engaging whole lysosome networks through biogenesis (left).

Collectively, our findings suggest a model in which microautophagy occurs in response to stress conditions that alter lysosomes but do not cause their rupture, as we and others have found that CASM-inducing stressors, including the ones used in our study like Monensin and Nigericin, do not induce rupture, yet lead to selective turnover within intact lysosomes. These “sub-rupture” stressors may induce CASM by altering the lysosomal pH, changing membrane tension, or altering other properties of the lysosomal membrane, as a result of modifying the osmotic environment of the lysosomal lumen. Microautophagy could conceivably sequester damaged portions of the limiting membrane onto ILVs to inhibit rupture, or could play a role in size control. Further studies uncovering additional regulators of this process may also shed light on its physiologic function. While we found that LC3 undergoes turnover, recent work has shown that all six orthologs of ATG8 in human cells contribute to regulate microautophagy, with a major contribution from an ATG8 subfamily called GABARAP, suggesting that further studies to quantify turnover of different ATG8 orthologs may be important. Overall, our study highlights that CASM has a dual ability to target both individual lysosomes for turnover and also whole lysosome networks through biogenesis (Figure 1). While transcriptional activation may necessitate that CASM affects the majority of lysosome in a network, microautophagy induction on the other hand could occur within even a single stressed lysosome, in a manner not predicted to affect transcription. CASM could therefore selectively impact individual lysosomes when stress is localized, and engage biogenesis when stress is more widespread across a lysosome network, a model that awaits further exploration.
